# Evaluation of the presence of SARS-CoV-2 in vaginal and anal swabs of women with omicron variants of SARS-CoV-2 infection

**DOI:** 10.3389/fmicb.2022.1035359

**Published:** 2022-11-08

**Authors:** Ding Liu, Yunfu Zhang, Dongfeng Chen, Xianhua Wang, Fuling Huang, Ling Long, Xiuhui Zheng

**Affiliations:** ^1^Department of Disease Prevention and Control, Daping Hospital, Army Medical University (Third Military Medical University), Chongqing, China; ^2^Department of Medical Affair and Research, Daping Hospital, Army Medical University (Third Military Medical University), Chongqing, China; ^3^Department of Gastroenterology, Daping Hospital, Army Medical University (Third Military Medical University), Chongqing, China; ^4^Department of Gynecology and Obstetrics, Daping Hospital, Army Medical University (Third Military Medical University), Chongqing, China

**Keywords:** coronavirus, SARS-CoV-2, omicron, vaginal fluid, anal swab

## Abstract

**Objectives:**

The study aimed to determine whether SARS-CoV-2 Omicron variant could be detected in the vaginal fluid and anal swabs of reproductive-aged and postmenopausal women infected with SARS-CoV-2 Omicron variant.

**Methods:**

Included in this study were 63 women who were laboratory confirmed as having SARS-CoV-2 Omicron variant infection and admitted to the responsible ward of Daping Hospital of at the National Exhibition and Convention Center(Shanghai) Makeshift Hospital from May 1–24, 2022.From them, vaginal and anal swabs were obtained with informed consent. The demographic and baseline clinical characteristics and the swab test results were analyzed.

**Results:**

The 63 included patients ranged in age from 18 to 72 years with a median of 47.71 ± 15.21 years. Of them, 38 women (60.3%) were in their reproductive years. Most of the participants (77.8%) were healthy without significant underlying diseases. Fourteen patients (22.2%) had asymptomatic infection and the remaining 49 (77.8%) had mild infection. The upper respiratory tract symptoms including cough (40/63.5%) and sore throat (18/28.6%)were the most common clinical manifestations of these mildly infected patients. Only 5 patients (7.8%) had gastrointestinal (GI) symptoms, including simple diarrhea in 4 patients, and diarrhea with vomiting in one patient. Pharyngeal,vaginal and anal swabs were collected simultaneously from all 63 patients 8–16 (mean 11.25 ± 2.23) days after SARS-Cov-2 Omicron variant infection. The vaginal swabs were negative for SARS-CoV-2 in all 63 patients, and the anal swabs were positive in 4 patients (6.5%). The overall median hospitalization duration was 16.73 ± 3.16 days.

**Conclusion:**

The results of the present study suggest that there is a low possibility of SARS-Cov-2 Omicron variant transmission *via* the digestive tract and vaginal fluid. The correlation between the GI symptoms and the presence of viral RNA in anal swabs is uncertain.

## Introduction

The Omicron variant of the novel coronavirus (COVID-19) has become the dominant epidemic strain in the world since it was first reported in South Africa in November 2021 (Vaughan, 2021).There has been an outbreak in Shanghai, China since March 2022, and the virus strain is Omicron variant BA.2. From March 1 to April 18, 2022, a total of 397,933 infected cases were reported, of which asymptomatic infections accounted for 93.06% of all infected people (Xian et al., 2022)，reflecting the characteristics of high infectivity and low pathogenicity of Omicron (Guo et al., 2022). Previous studies reported that they failed to detect severe acute respiratory syndrome coronavirus type 2 (SARS-CoV-2) in vaginal fluid swabs of patients with moderate to severe COVID-19 (Cui et al., 2020; Qiu et al., 2020; Uslu Yuvacı et al., 2021). However, viral nucleic acid was detected in patients with mild COVID-19, but only 3 positive cases were reported (Barber et al., 2021; Schwartz et al., 2021). Therefore, whether there is existence of infectious virions of SARS-CoV-2 in the vaginal fluid remains unclear. It was also reported that viruses could exist in the gastrointestinal (GI) tract for a long time and therefore anal swab detection could be used to judge the severity of the patient’s condition and guide epidemic prevention and control (Xu et al., 2020; Zhang et al., 2020). However, all these studies were carried out prior to the emergence of the current Omicron rampancy. With the virus mutation, the transmission ability, antibody resistance, immune escape and other abilities of the Omicron mutant have been enhanced (Cameroni et al., 2022), which can be transmitted through the mixture of aerosols and droplets. However, whether the virus will infect the female reproductive and GI tract has not been reported. As different strains may have different relative tropisms to tissues (Chertow et al., 2021), it is necessary to study female vaginal secretions and anal swabs to ascertain whether there are other transmission routes of SARS-CoV-2 Omicron variant beyond the respiratory system for the sake of providing a more scientific basis for its prevention and treatment.

The study aimed to determine whether SARS-CoV-2 Omicron variant was present in the vaginal fluid and anal swab of both reproductive-aged and postmenopausal women with SARS-CoV-2 Omicron variant infection.

## Materials and methods

### General information

Included in this study were 63 nonpregnant women who were laboratory confirmed as having SARS-CoV-2 Omicron variant infection and admitted to the responsible ward of Daping Hospital of at the National Exhibition and Convention Center(Shanghai) Makeshift Hospital from May 1 to May 24, 2022. They were diagnosed as asymptomatic or having mild infections based on the New Coronavirus Pneumonia Prevention and Control Program (9thedition) published by the National Health Commission of China (NHCC) (National Health Commission of China, 2022). Pharyngeal swab nucleic acid test was performed daily during hospitalization. Participants in pregnancy, menstruation and vaginitis symptoms were excluded from the study. Discharge criteria were as follows: the body temperature had returned normal for more than 3 days, the clinical symptoms of the respiratory tract were significantly ameliorated, and the CT values of SARS-CoV-2 nucleic acid N gene and ORF gene were ≥ 35 on pharyngeal swabs for two consecutive tests by qRT-PCR, the threshold value was 40, and the sampling time was at least 24 h apart.

This study was approved by the ethics committee of the said hospital (2022 No. 122–01), and written informed consent was obtained from all participants.

### Treatment measures

According to the NHCC New Coronavirus Pneumonia Prevention and Control Program (9^th^edition), asymptomatic and mild infections will be subjected to centralized isolation management. During isolation, symptomatic treatments and condition monitoring of the patients were performed. If the condition was aggravated, they would be transferred to designated hospitals for treatment.

### Reagents and instruments

The reagents and instruments use in this study were Sansure Natch96 semi-automatic extractor (Changsha, China); Hongshi SLAN PCR instrument(Shanghai, China); new coronavirus nucleic acid detection kit, nucleic acid extraction kit(Sansure Biotech, Changsha, China), and new coronavirus nucleic acid detection kit (Da An Gene Co.Ltd., Guangzhou, China). The fragments detected by the nucleic acid detection kits were ORF1ab/N genes, using the qRT-PCR method.

### Methods

#### Sampling method

Pharyngeal, vaginal and anal swabs were obtained simultaneously from all 63 patients between 8 and 16 days after onset of SARS-CoV-2 infection for SARS-CoV-2 nucleic acid. The pharyngeal swab test was performed by using the sterilized cotton swab rinsed with water to wipe the patient’s pharyngeal lateral wall and posterior pharyngeal wall several times and then sealing the swab in the sampling tube. The anal swab test was performed by using the sterilized cotton swab dipped with a small amount of 0.9% sterile sodium chloride solution, inserting it into the anal canal at a depth of 3 cm, rotating it for about 5 s, and sealing the swab in the sampling tube. The vaginal swab test was performed by inserting the sterilized cotton swab into the vagina in a 5–6 cm depth, rotating it for 4–5 rounds in about 7 s, and then sealing the swab in the sampling tube. All specimens were sent to the laboratory immediately after sampling.

SARS-CoV-2 nucleic acid detection: qRT-PCR was used to detect the open reading frame 1ab (ORF1ab) and nucleocapsid protein (N) in the SARS-CoV-2 genome.

Viral nucleic acid amplification Ct value detection method: the nucleic acid was extracted and placed in a reaction tube for amplification. The SARS-CoV-2 nucleocapsid protein N gene and the open reading frame OR-Flab were used as detection targets, and reverse transcription was performed at 50°C for 10 min (1 cycle); pre denaturation at 97°C for 1 min (1 cycle); denaturation at 97°C for 5 s; annealing or extension or fluorescence detection at 58°C for 30 s, 45 cycles as the amplification conditions.

Interpretation of the SARS-CoV-2 nucleic acid test results:(1) Target gene detection and interpretation. ①Negative: Ct value ≥35 or not detected; ②Positive: S-shaped amplification curve and Ct value<35.(2) Interpretation criteria for positive SARS-CoV-2 detection: ① Both targets (ORF lab, N) in SARS-CoV-2 in the same specimen were positive ② Re-sample and re-test for patients with positive single target. Single target positive, judged as positive.

### Statistical processing

In this study, SPSS 21.0 software was used to statistically process and describe the population distribution characteristics of the patients. The measurement data were described in the form of the mean ± standard deviation (x ± s), and the counting data were described in frequency (percentage, n (%)). Comparison of measurement data between two groups was performed by using two independent sample t-test, and comparison of counting data between two groups was performed by using chi square test and accurate probability method.

## Results

### Epidemiological characteristics

A total of 63 SARS-Cov-2 Omicron variant–infected female cases were collected, who ranged in age from 18 to 72 years with a median of 47.71 ± 15.21 years. Of them, 38 patients were in their reproductive period and 25 postmenopausal women. Twenty-six patients had received 3 vaccinations, 17 had received 2 vaccinations, one had received one vaccination, and 19 had not received any vaccination. Fourteen patients had underlying diseases, including hypertension in 8, type II diabetes mellitus (DM2) in 5, coronary heart disease (CHD) in 1, emphysema in 1, Hashimoto’s thyroiditis in 1, 1 week after abortion in 1, and knee joint replacement in 1. Four patients had two underlying diseases at the same time: hypertension+DM2 in 2, DM2 + CHD in 1, and hypertension+knee joint replacement in 1. The interval between onset of SARS-CoV-2 infection and sampling was 8–16 days with a mean of 11.25 ± 2.23 days **(** Table 1 **)**.

**Table 1:** Demographic and clinical characteristics of the patients.

Item	Total (*n* = 63)	Asymptomatic (*n* = 14)	Mild infection (*n* = 49) (%)	*p*-value^a^
Age (mean ± SD), years	47.71 ± 15.21	50.86 ± 16.28	46.82 ± 14.94	0.385
Status of menopause, *n* (%) Postmenopausal	25(39.7)	7(50.0)	18(36.7)	0.371
Reproductive-aged	38(60.3)	7(50.0)	31(63.3)	
				
Vaccination, *n* (%) Vaccination (2–3 shots)	43(68.3)	8(57.1)	35(71.4)	0.492
Unvaccinated or one shot	20(31.7)	6(42.9)	14(28.6)	
Underlying diseases status, *n* (%)				0.240
None	49(77.8)	13 (92.9)	36 (73.5)	
Combined underlying diseases	14(22.2)	1(7.1)	13(26.5)	
Chronic hypertension, *n* (%)	8(12.7)	0(0.0)	8 (16.3)	0.245
DM2, *n* (%)	5(7.9)	0(0.0)	5 (10.2)	0.578^c^
Others, (%)	5(7.9)	1(7.1)	4 (8.2)	0.258^c^
anal swabs, *n* (%)	4(6.5)	0(0.0)	4(8.3)	0.566^c^
Hospitalization (mean ± SD),days	16.73 ± 3.16	15.79 ± 3.47	17.00 ± 3.06	0.208^b^
Interval between infectiononset and sampling (mean ± SD), days	11.25 ± 2.23	11.14 ± 2.51	11.29 ± 2.17	0.834^b^

^a^chi-square test, ^b^independent-sample t test, ^c^Fisher exact test.

### Clinical symptoms

Among the 63 patients, 14 (22.2%) were asymptomatic and 49 (77.8%) had mild infections. The main clinical symptoms were cough, sore throat, and fever (>38.0°C), and may have myalgia, diarrhea, loss of taste, and fatigue. Other symptoms included chest tightness, nasal congestion, runny nose, hyposmia and chills (Table 2).

**Table 2:** Clinical symptoms.

Clinical symptoms	*n* (%)
Mild disease: Cough	40 (63.5)
Sore throat	18 (28.6)
Fever (>38°C)	10 (15.9)
Muscle soreness	7 (10.9)
Diarrhea	5 (7.9)
Loss of taste	4 (6.3)
Fatigue	3 (4.8)
Chest tightness	3 (4.8)
Stuffy nose	2 (3.2)
Decreased smell	2 (3.2)
Runny nose	1 (1.6)
Chills	1 (1.6)
Vomiting	1 (1.6)
Total	49 (77.8)
Asymptomatic	14 (22.2)

### Nucleic acid test results of specimens

Of the 63 patients, pharyngeal swab test was positive in 54 patients (85.7%), negative in 9 patients (14.3%), Among whom, 6 were asymptomatic and 3 had mild symptoms; anal swab test was positive in 4 patients (6.35%), all of whom had mild symptoms, only one had GI symptoms with diarrhea. Two had underlying diseases with well controlled hypertension, and 2 patients were healthy. Vaginal secretion test was negative in all patients.

## Discussion

SARS-COV-2 is a ribonucleic acid (RNA) virus with high mutation potential. The virus adapts to the new host through “mutation-selection-adaptation” (Cameroni et al., 2022), which means that the virus is a mutant strain with a strong replication ability but does not cause disease for most hosts; rather it has the opportunity to be selected in the process of popularity and rises to the main epidemic strain. The host cells actively capture the virus through receptors in the process of SARS-COV-2 infection. With the evolution of the virus, the affinity of the virus and host receptor ACE2 gradually improves, resulting in further reduction in the concentration of virus required for infection. Therefore, a stronger spreading ability and weaker pathogenicity should be expected as the evolution direction of SARS-COV-2 (Cao, 2020; Liu et al., 2021; Cameroni et al., 2022).

SARS-COV-2 has been found in nasopharyngeal secretion, feces, urine, semen, and tear since the outbreak of COVID-19 (Wang et al., 2020). Previous studies on the GI tract mostly focused on moderate-to severe-disease patients, in whom SARS-CoV-2 RNA has been detected in 40–85% fecal samples (Brooks and Bhatt, 2021), indicating that SARS-CoV-2 viral RNA can be detected in feces nearly as frequently as that in respiratory secretions. In addition, the positive rate of nucleic acid detection *via* anal swabs is related to the severity of the disease, and therefore it can be used as a supplement to the detection of respiratory specimens (Xu et al., 2020; Zhang et al., 2020). Natarajan et al. (2022) first studied the feces of patients with mild to moderate COVID-19, and found that fecal SARS-CoV-2 RNA was detected in 49.2% of the participants within the first week of diagnosis. With the extension of time, the positive rate gradually decreased, and the viral RNA was still detected in the feces of 3.8% patients 7 months after the diagnosis. They found that viral RNA in feces was the extended presence and fecal viral RNA shedding was correlated with GI symptoms. Their study is valuable for inferring population-level prevalence of COVID-19 from wastewater studies. For the study of SARS-CoV-2 in the female reproductive tract, no viral RNA was detected in vaginal fluid in patients with severe infection (Cui et al., 2020; Qiu et al., 2020; Uslu Yuvacı et al., 2021). But Schwartz et al. (2021) detected the SARS-CoV-2 virus in the vaginal secretions in 2 out of 35 women aged 21 and 86 years respectively, and both patients had mild symptoms. Sampling was conducted from the patients on day 6 and 11 after infection, respectively. Barber et al.[8]described 35 non-pregnant women with mild to moderately severe disease. SARS-CoV-2 was detected in one post-menopausal patient aged 60 years. Vaginal secretion sampling was performed on the same day of admission and diagnosis of COVID-19，In addition to the 21-year-old healthy woman, the other two patients have a variety of chronic diseases. Therefore, further clarifying the situation of the virus in the reproductive tract will have great significance for determining the risk of sex transmission and mother to child transmission during childbirth.

The Omicron variant is substantially mutated compared to any previously described SARS-CoV-2 isolates. It includes 37 substitutions of residues in the spike protein, 15 of which are clustered in the receptor-binding domain (RBD) (Cameroni et al., 2022), the key position of the ACE-2 receptor-cell interaction[19]and the main target of neutralizing antibodies after infection or vaccination (Liu et al., 2021). A study (Tian et al., 2022) reported that the Omicron RBD had 2.4-fold increased binding affinity to human ACE2 as compared with Wuhan-Hu-1 (Cameroni et al., 2022). The infectivity of Omicron might be more than 10-fold high as that of the original strain and approximately twice high as that of Delta. In the sera of volunteers with 3 vaccinations, the neutralizing antibody titer of the Omicron variant and the Delta variant reduced by 16.5-fold and 3.3-fold, respectively, as compared with that of the original strain (Wang et al., 2022). Therefore, while the gene mutation of the Omicron variant enhances the transmission ability, the ability to escape antibodies is also enhanced, which is the new transmission characteristic of the Omicron variant. To effectively prevent the spread of the virus, it is necessary to determine whether there are other different routes of transmission.

To answer this question, we detected nucleic acid simultaneously in pharyngeal, vaginal and anal swabs in 63 non-pregnant infected women at day 8–16 after the onset of SARS-CoV-2 infection, and found that the positive rate of pharyngeal swabs was 85.7%, positive anal swabs were detected in only 4 (6.35%) of the 63 included women, which is far lower than previous research[9、10、18], indicating that the ability of SARS-Cov-2 Omicron variant to invade the digestive tract is limited and the possibility of fecal oral transmission is low. No SARS-Cov-2 was detected in vaginal fluid. Our findings are supported by previous reports (Cui et al., 2020; Qiu et al., 2020; Uslu Yuvacı et al., 2021). But Schwartz and Barber reported 3 positive cases in the vaginal fluid (Barber et al., 2021; Schwartz et al., 2021), for which there are several possible explanations. First, although the binding affinity between the Omicron variant and Angiotensin-converting enzyme 2(ACE2) receptors is enhanced (Cameroni et al., 2022; Tian et al., 2022), the expression of the SARS-COV-2 ACE2 receptor in the vaginal tissue is low (Dimitrov, 2003; Uhlén et al., 2015; Morelli et al., 2021). it is possible that the SARS-Cov-2 Omicron variant does not seem to enter the vaginal fluid. Second, the incidence of viremia in patients with COVID-19 is low (Fajnzylber et al., 2020; Kim et al., 2020; Wang et al., 2020; Bwire et al., 2021; Hagman et al., 2021), and detection of the virus is feasible only in the viremia stage of the disease, indicating that the sampling time will affect the positive results. Additionally, this may be related to the Omicron inherent biology, the immune status of the host due to underlying disorders, natural immunization, vaccination, the duration of the virus invasion, and the sample size (Morelli et al., 2021).

The clinical characteristics of the 63 patients in our series were preliminarily analyzed, who ranged in age from 18 to 72 years with a median of 47.71 ± 15.21 years. The proportion of patients with mild infection was significantly higher than that of patients with asymptomatic infection(49/77.8% vs. 14/22.2%). The severity of SARS-Cov-2 infection depends on population characteristics, strain characteristics, and vaccination (Chen et al., 2022). Our statistical analysis showed that age, the menopausal status, underlying diseases, length of hospitalization, and other factors had no significant impact on symptoms (asymptomatic vs. mild) after infection. This may be due to the relatively small number of the participants and the relatively long hospitalization duration as compared with the mean hospitalization duration of 7 days. In our study, 43 patients (68.3%) were vaccinated more than twice, and the incidence of mild disease had no significant difference from that of the uncompleted vaccination group. The four patients with positive anal swabs all received more than 2 vaccinations, suggesting that the gene mutation of the Omicron variant may weaken the effectiveness of SARS-Cov-2 vaccine. However, another study (Puhach et al., 2022) showed that vaccination still had a certain protective effect in reducing the incidence of severe diseases, the risk of death and transmission. In the current epidemic in Shanghai, asymptomatic infections accounted for 93.06%, low severe infections and low mortality also supported the above view.

The most common clinical manifestations of mild infected patients were upper respiratory tract symptoms such as cough and sore throat, which may be related to the fast replication rate of the Omicron variant in bronchial epithelial cells, and the weak replication rate in alveolar cells (Hui et al., 2022). Of the five patients with diarrhea, the anal swab test was positive in only one patient, suggesting that the correlation between the GI symptoms and the presence of viral RNA in the GI tract remains uncertain, and the GI symptoms may largely be the manifestation of systemic symptoms after infection.

This study has some limitations. First, the samples were only extracted from patients 8–16 days after the infection, and dynamic research on other periods is absent. It cannot fully explain the characteristics of virus transmission through the lower genital tract during the whole infection process. In addition, although asymptomatic patients and those with mild infection could be complementary to previous studies to some extent, the study did not include patients with other subtypes of Omicron variants. More comprehensive epidemiological studies with larger sample sizes are required to further verify the findings and conclusions in this study.

In summary, our results demonstrated that among asymptomatic and mildly infected non-pregnant women, the Omicron variant has limited dissemination capacity through the GI tract, and does not possibly spread through the lower genital tract. However, this conclusion needs to be confirmed by further in-depth and comprehensive research. At present, the number of patients infected with Omicron mutants continues to increase globally. It seems that the variation and transmission of SARS-CoV-2 has reached normalcy. This study will not only give guidance to choosing the approach of laboring for pregnant women with Omicron infection, but also provide useful references for the prevention and control of COVID-19.

## Data availability statement

The raw data supporting the conclusions of this article will be made available by the authors, without undue reservation.

## Ethics statement

The studies involving human participants were reviewed and approved by Ethics Committee of Daping Hospital, Army Medical University. The patients/participants provided their written informed consent to participate in this study.

## Author contributions

XZ had full access to all the data in the study and takes responsibility for the integrity of the data and the accuracy of the data analysis and supervision. XZ and DL: study concept and design. DL and YZ: acquisition of data and drafting of the manuscript. DL, YZ, DC, XW, FH, and LL: analysis and interpretation of data and statistical analysis. DL, YZ, and XZ: critical revision of the manuscript for important intellectual content. All authors contributed to the article and approved the submitted version.

## Funding

The work was supported by the Talent Innovation Ability Training Plan of the Army Medical Center (2019CXLCC014); Chongqing Natural Science Foundation (cstc2021jcyj-msxmX1027); Chongqing Medical Scientific Research Project (Joint Project of Chongqing Health Commission and Science and Technology Bureau) (2019ZDXM009).

## Conflict of interest

The authors declare that the research was conducted in the absence of any commercial or financial relationships that could be construed as a potential conflict of interest.

## Publisher’s note

All claims expressed in this article are solely those of the authors and do not necessarily represent those of their affiliated organizations, or those of the publisher, the editors and the reviewers. Any product that may be evaluated in this article, or claim that may be made by its manufacturer, is not guaranteed or endorsed by the publisher.
